# PDX-Derived Ewing’s Sarcoma Cells Retain High Viability and Disease Phenotype in Alginate Encapsulated Spheroid Cultures

**DOI:** 10.3390/cancers13040879

**Published:** 2021-02-19

**Authors:** Giacomo Domenici, Rodrigo Eduardo, Helena Castillo-Ecija, Gorka Orive, Ángel Montero Carcaboso, Catarina Brito

**Affiliations:** 1 iBET, Instituto de Biologia Experimental e Tecnológica, Apartado 12, 2781-901 Oeiras, Portugal; giacomo.domenici@ibet.pt (G.D.); rodrigo.eduardo@ibet.pt (R.E.); 2 Instituto de Tecnologia Química e Biológica António Xavier, Universidade Nova de Lisboa, Av. da República, 2780-157 Oeiras, Portugal; 3 Pediatric Hematology and Oncology, Hospital Sant Joan de Deu, Institut de Recerca Sant Joan de Déu, Passeig Sant Joan de Déu 2, 08950 Barcelona, Spain; hcastillo@fsjd.org (H.C.-E.); amontero@fsjd.org (Á.M.C.); 4 NanoBioCel Group, Laboratory of Pharmaceutics, School of Pharmacy, University of the Basque Country UPV/EHU, Paseo de la Universidad 7, 01006 Vitoria-Gasteiz, Spain; gorka.orive@ehu.es; 5 Biomedical Research Networking Centre in Bioengineering, Biomaterials and Nanomedicine (CIBER-BBN), Paseo de la Universidad 7, 01006 Vitoria-Gasteiz, Spain

**Keywords:** Ewing’s Sarcoma, PDX-derived cells, tumour spheroids, 3D cultures, alginate, drug assays

## Abstract

**Simple Summary:**

Ewing’s Sarcoma (ES) is the second most frequent bone tumour in children and young adults, with very aggressive behaviour and significant disease recurrence. To better study the disease and find new therapies, experimental models are needed. Recently, patient-derived xenografts (PDX), obtained by implanting patient tumour samples in immunodeficient mice, have been developed. However, when ES cells are extracted from the patient’s tumour or from PDX and placed on plasticware surfaces, they lose their original 3D configuration, cell identity and function. To overcome these issues, we implemented cultures of PDX-derived ES cells, by making them aggregate to form ES cell spheroids and then encapsulating these 3D spheroids into a hydrogel, alginate, to stabilize the culture. We show that this methodology maintained ES cell viability and intrinsic characteristics of the original ES tumour cells for at least one month and that it is suitable for study the effect of anticancer drugs.

**Abstract:**

Ewing’s Sarcoma (ES) is the second most frequent malignant bone tumour in children and young adults and currently only untargeted chemotherapeutic approaches and surgery are available as treatment, although clinical trials are on-going for recently developed ES-targeted therapies. To study ES pathobiology and develop novel drugs, established cell lines and patient-derived xenografts (PDX) are the most employed experimental models. Nevertheless, the establishment of ES cell lines is difficult and the extensive use of PDX raises economic/ethical concerns. There is a growing consensus regarding the use of 3D cell culture to recapitulate physiological and pathophysiological features of human tissues, including drug sensitivity. Herein, we implemented a 3D cell culture methodology based on encapsulation of PDX-derived ES cell spheroids in alginate and maintenance in agitation-based culture systems. Under these conditions, ES cells displayed high proliferative and metabolic activity, while retaining the typical EWSR1-FLI1 chromosomal translocation. Importantly, 3D cultures presented reduced mouse PDX cell contamination compared to 2D cultures. Finally, we show that these 3D cultures can be employed in drug sensitivity assays, with results similar to those reported for the PDX of origin. In conclusion, this novel 3D cell culture method involving ES-PDX-derived cells is a suitable model to study ES pathobiology and can assist in the development of novel drugs against this disease, complementing PDX studies.

## 1. Introduction

Ewing’s Sarcoma (ES) is a rare kind of tumour most common in adolescents and young individuals. It affects soft tissues and bones, and it is generally characterized by a high grade and malignancy [1]. Even though most ES patients are treated with chemotherapy and tumour resection and radiotherapy achieve complete remission of their primary tumours, relapses are very common [2]. Recurrence in ES is commonly characterized by distant metastases in lung/bones, especially one to two years after initial diagnosis (70% of the cases), while late relapse at two to five years after first diagnosis has been sporadically reported [1]. ES, as well as other sarcomas [2,3,4], is typically characterized by the presence of chromosomal aberration [5]. For ES in particular, the most characterized ones are chromosomal translocations between the FET family of RNA-binding proteins (EWSR1, FUS and TAF15) and the ETS transcription factors FLI1 and ERG. These translocations generate potent oncogenic transcription factors that are considered the molecular drivers of the disease [2,6]. The most commonly found is the chimeric protein EWSR1-FLI1 t(11;22)(q24;q12), which is detected in almost 90% of the cases, while EWSR1-ERG t(21;22)(q22;q12) accounts for only 10% of the cases [7]. Targeted therapeutic approaches have been recently developed, such as drugs that work against the oncogenic protein EWSR1-FLI1 and the Insulin-like Growth Factor 1 Receptor (IGF-1R) [8,9], which are currently in clinical trials (clinical trial numbers: NCT02657005 and NCT02306161, respectively).

Over the years, ES has been mostly studied through the use of cell lines [10], as well as established patient-derived xenografts (PDX) [11,12]. Due to the general low availability of novel ES cases as well as the poor success rate in establishing novel cell lines [13,14], the total number of recognized ES cell lines amounts to around ten [15]. Adding to these drawbacks in the cell line development process, it is nowadays recognized that 2D cell cultures fail to represent the biological and molecular complexity that characterize tumours [16,17]. During the last decade, worldwide implementation of the in vivo PDX technology has enabled researchers to study various kinds of tumours in a more detailed manner and opened new venues for personalized therapy, even for sarcomas [18]. Nevertheless, economic and ethical issues related to animal research [19] raise the need for the development of other suitable methods to maintain ES cell cultures ex vivo. Such cultures would be more suitable for drug testing and high throughput screening purposes and could also be used to recapitulate human tumour microenvironment (TME), which is substituted with mouse stromal cells in the PDX models upon passage [20].

Novel ex vivo models can be implemented either directly from patient biopsies or from established PDX, with the aim of creating a complementary tool to develop novel drugs against ES or to better depict biological/molecular mechanisms, which stand behind ES aetiology and pathobiology. One opportunity is provided by using 3D cell models, based either on tumour spheroids or patient-derived organoids, which better recapitulate the complexity, cellular architecture and molecular cascades found in tumours in vivo, enabling the maintenance of tumour cells along with cells of the TME, such as fibroblasts or immune cells [21,22,23,24]. In the 3D sarcoma cell model field, the first attempt was in 1985: Bruland and colleagues generated cultures of multicellular spheroids from a variety of sarcoma patients, such as hemangiopericytoma and synovial sarcoma [25]. Sarcoma cells were successfully expanded as spheroids for one up to several weeks. Nevertheless, in this study ES was not addressed. Almost two decades later, Lawlor and colleagues reported the generation of ES spheroids kept in culture for up to 48 h and derived from established human ES cell lines [26]. In another study, ES spheroid cultures were successfully generated from ES cell lines but not from the primary cultures of ES cells [27]. Generation of ES spheroid cultures directly derived from patient material, either post-surgery or from established PDX, has been hindered due to the scarcity of primary ES material. Therefore, we proposed to establish primary 3D ES cultures from PDX mouse models developed at Hospital Sant Joan de Déu (HSJD) in Barcelona (Spain) [11,12]. We developed 3D ES spheroid cultures which were subsequently encapsulated in the inert biomaterial alginate, following a strategy previously developed by us [28,29]. Alginate is a biocompatible and biodegradable material [30] and we have previously shown that alginate encapsulation provides mechanical support to sustain cell spheroid structure with high cell viability and extracellular matrix (ECM) molecules secreted by the cells are deposited within the capsules [29]. Here, we show that alginate encapsulation followed by culture under agitation sustains ES spheroid cell viability and proliferation for up to at least one month of culture. ECM components were detected in encapsulated ES spheroids, as well as maintenance of EWSR1-FLI1 translocation. In addition, the model was challenged by drug treatments. Interestingly, we observed a reduced mouse cell contamination compared to original PDX and 2D counterpart cultures. In summary, in this study, we presented a novel ES 3D model that can be employed both to better understand ES biology and for drug screening, chemotherapy resistance and TME studies.

## 2. Materials and Methods

### 2.1. Animals

The research conducted with mice was approved by the local animal care and use committee (animal protocol number 135/11, approved by CEEA-UB (Comitè Ètic d’Experimentació Animal de la Universitat de Barcelona)) and was carried out in accordance with institutional and European guidelines (EU Directive 2010/63/EU) and the ARRIVE guidelines. ES PDX models were developed from patient biopsies at HSJD under a protocol approved by the Institutional Review Board. PDX tissues were maintained in mice by transplanting fresh PDX fragments (10–50 mm^3^) to the flanks of female athymic nude mice (Envigo, Barcelona, Spain).

### 2.2. Samples Processing

Briefly, PDX tissues were washed with PBS (Gibco, Thermo Fisher Scientific, Waltham, MA, USA), minced into small pieces of approximately 2–3 mm in diameter and digested with Collagenase Type II (125 U/mL, Worthington, 087001, Lakewood, NJ, USA) in the presence of Benzonase (30 U/mL, Merck Millipore, Darmstadt, Germany) for up to 30 min at 37 °C, under continuous stirring at 1000 RPM. The resulting single-cell suspensions were washed with PBS and centrifuged for 3 min at 300× *g*. Cells were cultivated directly or cryopreserved at −80 °C in FBS supplemented with 10% DMSO or in Bambanker (Fujifilm Wako Chemicals Europe GmbH, Neuss, Deutschland) for 24 h. Cells were subsequently stored in liquid nitrogen for long-term storage.

### 2.3. Cell Culture and Spheroid Encapsulation

ES cells were cultured in the following complete medium: RPMI media supplemented with 2 mM Glutamax, 10% (*v*/*v*) FBS, 1% (*v*/*v*) penicillin/streptomycin, 1 mM Sodium Pyruvate and 0.10 mM non-essential amino acids (all from Life Technologies, Waltham, 02451, MA, USA). Cell aggregation was carried out in static conditions by plating 3 × 10^6^ cells in ultra-low attachment plates (Cat. CLS3815 Corning, NY, USA). After three days of aggregation, the spheroid suspension was recovered by mild centrifugation at 50× *g* for 3 min and washed with PBS. The spheroids were resuspended and encapsulated in 1.1% (*w*/*v*) of Ultrapure Ca^2+^ MVG (UP MVG NovaMatrix, Pronova Biomedical, Sandvika, Norway), prepared in NaCl 0.9% (*w*/*v*) solution, as previously reported in our lab [29]. The microencapsulation was performed with a microencapsulator (VarV1, Nisco, Zurich, Switzerland) to generate beads with diameters of approximately 1 mm. Alginate polymerization was achieved in a 20 mM BaCl_2_ solution. The microcapsules were collected, washed three times in NaCl 0.9% (*w*/*v*) to remove BaCl_2_ residues and resuspended in 25 mL of complete medium. Subsequently, alginate-encapsulated spheroids were cultured in shake flasks (Corning) under continuous orbital agitation, following the method previously optimized for cancer cell lines [28,29]. Half of the culture medium was exchanged every three to four days.

### 2.4. Spheroid Retrieval from Alginate Capsules and Diameter Analysis 

Cultured spheroids were retrieved from alginate capsules using a chelating solution (10 mM HEPES, 100 mM EDTA, pH 7.4). Spheroid size was estimated by measuring Feret’s diameter through the open-source ImageJ software. Statistical analysis was undertaken through a non-parametric Mann–Whitney test for comparison of the aggregate diameter.

### 2.5. Metabolic Viability Assessment

Metabolic activity was assessed through resazurin reduction by using the PrestoBlue™ Viability Reagent reduction assay (Cat. #A13262, Life Technologies), following the manufacturer’s instruction. The samples were incubated with the reagent for one hour at 37 °C and the fluorescence was read at 560 nm excitation and 590 nm emission in an Infinite^®^ 200 PRO microplate reader (NanoQuant, Tecan Trading AG, Männedorf, Switzerland). Metabolic activity was measured three to four days post-encapsulation and repeated once a week, for up to one month in culture. Results are shown as the fold change in metabolic activity relative to the first day of measurement (ES-6, -12, -16: *n* = 3, ES-2, -11: *n* = 2). The non-parametric Kruskal–Wallis test was performed for statistical analysis.

### 2.6. Cell Viability Analysis

Cell viability was assessed through a fluorescent-based membrane integrity assay to discriminate live from dead cells. Microcapsules were incubated with 10 μg/mL of the cell-permeant compound fluorescein diacetate (FDA; Sigma-Aldrich, St. Louis, MO, USA) and 1 μM of the cell-impermeant DNA probe TO-PRO^®^ 3 (Invitrogen, Waltham, 02451, MA, USA) and observed under a fluorescence microscope (DMI6000, Leica Microsystems GmbH, Wetzlar, Germany). Cells that accumulated and metabolized the green, fluorescent product of FDA were considered live and cells stained with TO-PRO^®^ 3 were considered dead.

### 2.7. Cell Proliferation Analysis

To assess cell proliferation, we monitored DNA synthesis throughout the culture. ES spheroids (cultured alone or within alginate microcapsules) were sampled from shake flasks at specific time points. ES spheroids were recovered from capsules by using a chelating solution (10 mM HEPES, 100 mM EDTA, pH 7.4) and recovered by centrifugation at 50× *g* for 1 min. Pellets were resuspended in 1 mL of DNAse/RNAse-free water (Invitrogen) and stored at −80 °C until analysis. Once all samples were collected, they were subjected to 30 min of ultrasounds to lyse cells and release DNA. Cell proliferation was measured by the amount of dsDNA present in the samples using the Quant-iT™ PicoGreen^®^ dsDNA Assay Kit (Invitrogen), following the manufacturer’s instructions. dsDNA quantification was normalized by the PrestoBlue^TM^ assay performed in capsules before the recovery of the spheroids. Data are presented as the fold change of the dsDNA content compared to day 0, set as 1. The non-parametric Kruskal Wallis test was performed for statistical analysis.

### 2.8. Exposure to Chemotherapeutic Drugs

Encapsulated and non-encapsulated spheroids were cultured for two weeks in shake flasks before proceeding to drug exposure. Subsequently, spheroids were distributed in 12-well plates and the PrestoBlue™ Viability Reagent reduction assay (Cat. #A13262, Life Technologies) was performed according to the manufacturer’s instruction. Subsequently, spheroids were exposed to the chemotherapeutic drugs SN-38 (the active metabolite of irinotecan) and docetaxel (Carbosynth, Comptom, UK), in a nano-range of concentration (0–100 nM). Both drugs were diluted in DMSO, which served as vehicle control. Cells were exposed to drugs for seven days, adding fresh medium with drugs after three to four days. Then, the resazurin reduction ability was measured again and each well was normalized to the initial measurement. Results are shown as the fold change in metabolic activity compared to the activity of cells exposed to the vehicle, which was set as 1. The encapsulated spheroids were: ES-2, -11: *n* = 2, ES-6, -12, -16: *n* = 3; non-encapsulated spheroids: ES-6, -12: *n* = 3 ES-16: *n* = 2. The analysis was complemented by FDA/TO-PRO^®^ 3 staining for visualization of live/dead cells by microscopy, as described above.

### 2.9. RNA Extraction, RT-qPCR Analysis, and Detection of EWSR1-FLI1 Expression

RNA from 2D and 3D ES cultures, as well as HepG2 and MCF-7 cells, were extracted with a High Pure RNA Isolation Kit (Cat. #11828665001, Roche, Basel, Switzerland) and stored at −80 °C until use. cDNA synthesis was performed with a Transcriptor High Fidelity cDNA synthesis kit (Cat. #05091284001, Roche). RNA extraction and cDNA synthesis were both performed according to the manufacturer’s instructions. RT-qPCR was performed using SYBR-Green (SYBR Green I Master Kit, Roche), in LightCycler 480 equipment (Roche). Gene expression was determined with the comparative CT method (2^−ΔΔCT^), using *RPL22* and *36B4* [29,31] expression as control (*n* = 2). The sequences of the primers used in this study are listed in Table S2. *EWSR1-FLI1* translocation in ES cultures was assessed by RT-qPCR, using primers that amplify the fusion transcript between *EWSR1* and *FLI-1* [5]. RT-qPCR amplification products were separated by electrophoresis (2% agarose gel, Cat. #MB02703, NZYTech, Lisbon, Portugal) and amplicons were visualized using the RedSafe Nucleic Acid Staining Solution (Cat. #21141, Intron Biotechnology, Seongnam, South Korea). The same cDNAs were used to amplify 36B4 (84 bp) and examined for amplicon size comparison. A negative control (water, H_2_O) for EWSR1-FLI1 was run in parallel.

### 2.10. Mouse Cells Contamination Analysis

DNA was extracted from original ES PDX cells after one-month culture in alginate by using the DNAeasy Blood and Tissue Kit (Cat. #69504, Quiagen, Germantown, MD, USA), following the manufacturer’s instructions. The amount of human and mouse DNA in the 3D cultured samples versus the original PDX cell was indirectly estimated by qPCR using specific human and mouse *PTEGR2* primer sequences used in a published study [32]. Real-time PCR was performed using SYBR-Green (SYBR Green I Master Kit, Roche, Basel, Switzerland) in a LightCycler 480 (Roche, Basel, Switzerland). Data were analysed by comparing human and mouse Ct *PTEGR2* values. For ES-6 and ES-12, mouse *PTEGR2* Ct values were subtracted from human *PTEGR2* Ct values to obtain ΔΔCt values. Subsequently, 2^−ΔΔCt^ values were calculated and multiplied by 100 to obtain the percentage of mouse genome. Amplification of the mouse ribosomal 36B4 mRNA was assessed in four different ES cultures (2D and 3D) using a mouse-specific primer. Primer BLAST analysis was used to assess the specificity of the primers. Amplification in mouse but not in human DNA was further assessed by using mouse NIH-3T3 fibroblasts as positive control and the liver hepatocarcinoma cells HepG2 as negative control. Welch’s *t*-test was performed for statistical analysis.

### 2.11. Hypoxia Detection

Environmental hypoxia was detected with the fluorescent probe Image-iT™ Green Hypoxia Reagent (Invitrogen, Catalogue number: I14833), a probe which becomes fluorescent when live cells are in an environment with low atmospheric oxygen levels (≤5%), following the manufacturer’s instructions. Briefly, microencapsulated ES spheroids and MCF-7 monolayers were incubated with 5 µM of Image-iT^TM^ Green for 1 h and imaged in a Leica DMI6000 inverted microscope (Leica Microsystems GmbH, Wetzlar, Germany). Carbonic anhydrase IX (*CAIX)* mRNA expression was assessed through RT-qPCR, as described above. As positive and negative controls of the hypoxia environment and signalling activation, the breast cancer cell line MCF-7 was either exposed to 800 µM CoCl_2_ for 24 h, cultured under 3% O_2_ (hypoxia controls) or cultured in 5% CO_2_ in air as normoxia control.

### 2.12. Immunofluorescence Analysis

Alginate capsules were collected from shake flasks and processed for immunofluorescence as described before [29]. Briefly, fixation was performed with 4% paraformaldehyde for 10 min at room temperature. Subsequently, alginate capsules were dehydrated overnight in 30% sucrose. After removing sucrose excess, the capsules were encapsulated in Tissue-Tek^®^ O.C.T.™ Compound (Sakura, Alphen aan den Rijn, Netherlands and frozen at −80 °C until use. Cryosections with a thickness of 10 µm were generated in a cryostat (Cryostat CM 3050 S, Leica). The cryosections were permeabilized with 0.1% (*v*/*v*) Triton X- 100 (Sigma-Aldrich) for 10 min and blocked with 0.2% (*w*/*v*) fish-skin gelatine (FSG; Sigma-Aldrich) for 30 min. Primary and secondary antibodies were prepared in 0.125% (*w*/*v*) FSG in PBS and incubated for 2 or 1 h, respectively. The samples were mounted in Prolong^®^ Gold antifade reagent containing DAPI (Life Technologies). The primary antibodies used were anti-Collagen type I (AB 34710, Abcam, Cambridge, UK), anti-Collagen type IV (AB 6586, Abcam), anti-Fibronectin (AB2413, Abcam), anti-Laminin (MAB19562, Millipore), anti-Vimentin (V6389, Sigma, Saint Louis, MO, USA) and anti-Ki67 (AB16667, Abcam). Secondary antibodies (conjugated with Alexa488, Invitrogen) were used in accordance with the primary antibody. Samples were visualized using a fluorescence microscope (DMI6000, Leica). The 2D cultures were fixed in 4% paraformaldehyde for 10 min at room temperature and immunofluorescence procedures were followed as above, without the sucrose dehydration step.

### 2.13. Flow Cytometry Analysis

ES spheroids were retrieved from alginate capsules using a chelating solution as indicated above. Spheroids were washed with PBS, centrifuged at 300× *g* for 5 min, resuspended in TrypLE™ Express Enzyme 1X (Gibco) and incubated at 37 °C. Every 2 min, spheroids were gently pipetted until a single cell suspension was obtained. Cells were washed once in PBS and incubated for 30 min on ice with anti-CD90/Thy-1 antibody conjugated with FITC (Cat. 555595, Biolegend, San Diego, CA, USA) or with control IgG-FITC (Cat. 555578, Biolegend), in a PBS-1% BSA solution. Then, cells were washed twice with PBS-1% BSA solution, filtered through CellTrics^®^ 30 µm filters (Sysmex, Kobe, Japan) to obtain a single cell solution and analysed in a CyFlow space (Sysmex). Data and histograms were generated in FlowJo software (Version 10, BD, Franklin Lakes, NJ, USA).

## 3. Results

### 3.1. PDX-Derived ES Cell Spheroid Cultures Sustain Cell Proliferation and Reduce Mouse Cell Contamination

**Figure 1:**
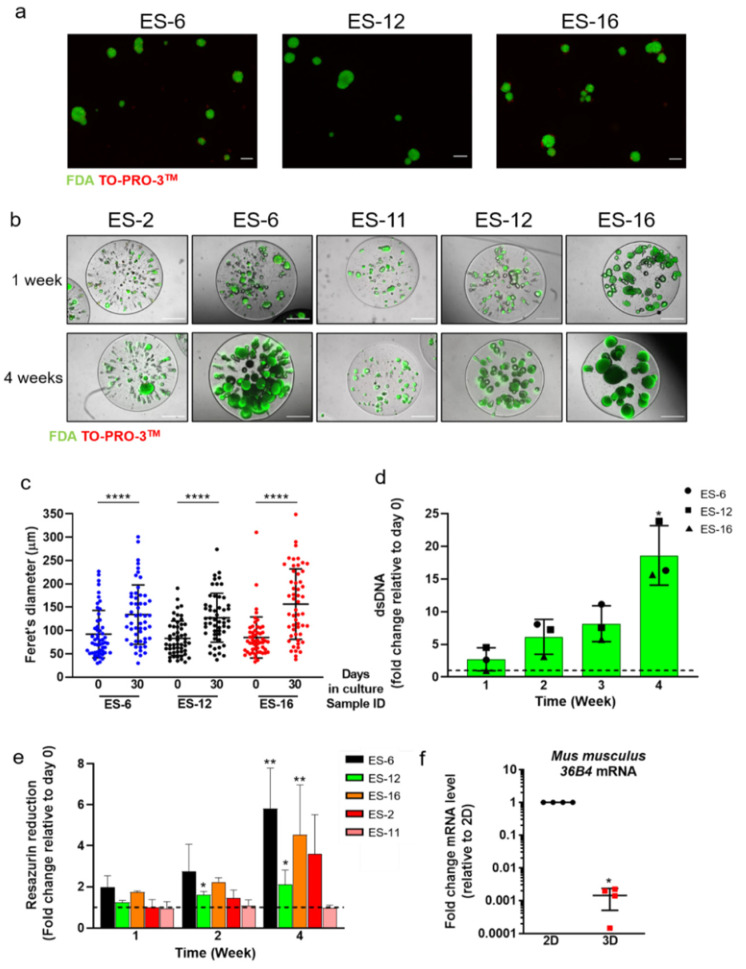
Cell viability and proliferation in encapsulated Ewing’s Sarcoma (ES) spheroid cultures. (**a**) ES spheroids formed from single cell suspensions of three distinct ES patient-derived xenografts (PDX); live cells: FDA; dead cells: TO-PRO-3^TM^. Scale bar: 100 µm. (**b**) Representative images of encapsulated spheroid cultures derived from five distinct ES-PDX along culture time (after one and four weeks). Scale bar: 500 µm. (**c**) ES spheroid size distribution (mean ± SD) after aggregation (day 0) and after 30 days of encapsulated culture; statistical analysis was performed with the non-parametric Mann–Whitney test (**** *p* < 0.0001). (**d**) dsDNA amount along encapsulated spheroid cultures derived from three distinct ES-PDX, shown as the fold change at each culture time relative to day 0 (set as 1, dashed line). Statistical analysis was performed with the non-parametric Kruskal–Wallis test (* *p* = 0.0250). (**e**) Resazurin reduction along encapsulated spheroid cultures derived from five distinct ES-PDX, shown as the fold change at each culture time relative to day 0 (set as 1, dashed line). ES-6, 12, 16, 11: *n* = 3, ES-2: *n* = 2. Statistical analysis was performed with the non-parametric Kruskal–Wallis test (* *p* < 0.05; ** *p* < 0.001). (**f**) Mouse *36B4* mRNA level in encapsulated spheroid cultures derived from four distinct ES-PDX, shown as the fold change in mRNA level relative to 2D cultures (set as 1, dashed line). Statistical analysis was performed with Welch’s *t*-test (* *p* < 0.0001).

Five ES-PDX samples, named with the codes HSJD-ES-002, -006, -011, -012 and -016, were obtained fresh from the flanks of athymic nude mice; HSJD-ES-002 and -006 were derived from the same ES patient, at diagnosis and relapse, respectively, and the remaining samples from three distinct ES patients (HSJD-ES-11, -12, -16). Clinical details of the original biopsies and PDX engraftment times are shown in Table S1. Tissues were mechanically and enzymatically dissociated and plated in ultralow-attachment plates. The impaired adherence to the plastic promoted rapid self-aggregation of the cells [25], generating rounded and compact multicellular spheroids (Figure 1a). After encapsulation of spheroids in alginate, cultures were kept under agitation for up to one month; tumour spheroids within capsules maintained high cell viability (Figure 1b). The Feret’s diameter of spheroids, determined before encapsulation and after one month of culture, increased in the three independent PDX cultures evaluated: HSJD-ES-006 (ES-6, from 92.2 ± 50.9 to 134.2 ± 63.7 μm), HSJD-ES-012 (ES-12, from 83.4 ± 37 to 127.4 ± 52.4 μm) and HSJD-ES-016 (ES-16, from 85.1 ± 44.0 to 156.6 ± 75.3 μm), as illustrated in Figure 1c. Active proliferation within encapsulated spheroids was confirmed by the detection of Ki67 positive cells (Figure S1a). In non-encapsulated ES spheroid cultures, we observed the formation of very large structures, probably due to spheroid fusion, which maintained spherical morphologies (Figure S1b). Despite the presence of viable cells, stained with FDA, we also observed the generalized presence of TO-PRO-stained cell debris, suggesting increased cell death. The latter may have been a consequence of the shear stress due to continuous agitation or due to nutrient and gas diffusion limitations in larger spheroids (Figure S1b). Interestingly, in non-encapsulated cultures, dsDNA amount did not increase over time (Figure S1c), contrary to what was observed for encapsulated ES spheroids (Figure 1d). During the one-month culture under orbital shaking, ES spheroids retained metabolic activity, as shown by the PrestoBlue™ Viability Reagent reduction assay (Figure 1e), displaying a tendency for a time-dependent increase for the samples ES-6, -16, -12 and -2, while for ES-11 it remained constant. Importantly, the structure of the alginate capsules remained stable throughout the one month of culture (Figure 1b). Encapsulation and maintenance of ES spheroids was successful for all the PDX tested.

Mouse cell contamination is a very well-known drawback of PDX-derived cultures [33,34]. Therefore, we assessed mouse cell contamination in our 3D ES-PDX-derived cultures by quantifying the mRNA level of the mouse acidic ribosomal phosphoprotein P0 (also known as *36B4*), using mouse-specific primers that do not amplify any target sequence in human cells (Figure S1d). The amount of mouse *36B4* mRNA was drastically reduced in 3D cultures compared to the monolayer culture counterparts (Figure 1f). In addition, amplification of a mouse- and human-specific genomic sequence of *PTGER2* [32] indicated that the level of mouse genome was reduced in 3D culture (0.07 ± 0.015%) compared to the original PDX (6.45 ± 1.43%) (Figure S1e). This suggests that the 3D culture method applied here favours the growth of human cells over mouse cells.

Finally, we evaluated the presence of hypoxia within tumour spheroids by assessing the *CAIX* expression of. The gene contains a hypoxic response element (HRE) under the control of the hypoxia-inducing factor alpha (HIF-1α) and therefore its transcription is induced under hypoxic conditions [35]. The level of expression of *CAIX* was unchanged in 3D relative to 2D ES cultures (Figure S1f). As a control, we detected an increase in expression of *CAIX* in the MCF-7 breast cancer cells exposed to CoCl_2_ (a chemical hypoxia inducer) and to 3% O_2_ (Figure S1g). Moreover, we evaluated environmental hypoxia by using a probe which becomes fluorescent when live cells are in an environment with low atmospheric oxygen levels (≤5%). Green fluorescence was detected in MCF7 cells cultured under 3% O_2_ but not in encapsulated ES spheroids (Figure S1h). The data suggest that there was no impairment of oxygen diffusion within the encapsulated 3D ES spheroids cultured under agitation.

### 3.2. ES Cells in Encapsulated Spheroid Cultures Retain EWSR1-FLI1 Translocation

**Figure 2:**
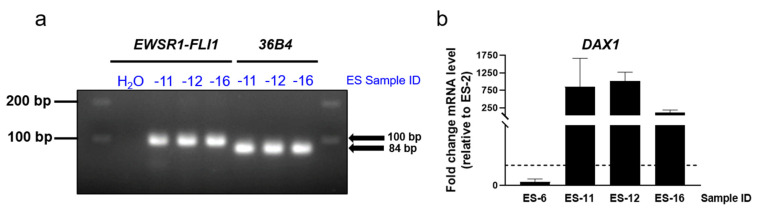
*EWSR1-FLI1* and *DAX1* mRNA expression in encapsulated ES spheroids. (**a**) DNA agarose gel showing *EWSR1-FLI1* and *36B4* amplification products in encapsulated spheroids derived from three distinct ES-PDX samples. (**b**) *DAX1* mRNA level in encapsulated spheroids derived from three distinct ES-PDX, shown as the fold change in mRNA level compared to ES-2, set as 1 (*n* = 2).

Having established that the method shown here maintained ES cell viability and proliferation, we aimed to confirm that ES cell identity was retained. One of the key hallmarks of ES is the presence of a chromosomal translocation between *EWSR1* and *FLI1*, which generates a chimeric transcription factor that exerts profound oncogenic effects in ES cells [2]. Therefore, it was important to establish whether this translocation product was maintained in our 3D cultures. We applied an RT-qPCR-based approach, using a primer set that has been described to selectively amplify between *EWSR1* (exon 7) and *FLI1* (exons 1–5 or 1–6, according to the translocation breaking point) [5]. The *EWSR1-FLI1* transcript was amplified and the expected amplicon (100 bp) was confirmed (Figure 2a). The *36B4* amplification product (84bp) was run in parallel for size comparison and to confirm the cDNA quality. In addition, we detected the EWSR1-FLI1 target gene *DAX1* [36], strongly expressed in ES-12, ES-16 and ES-11 compared to ES-2 and ES-6 (Figure 2b; uncropped and unadjusted agarose gel image relative to Figure 2A are shown in Figure S4).

### 3.3. ES Cells in Encapsulated Spheroid Cultures Express Typical Mesenchymal Cell Markers and Secrete ECM Components

**Figure 3:**
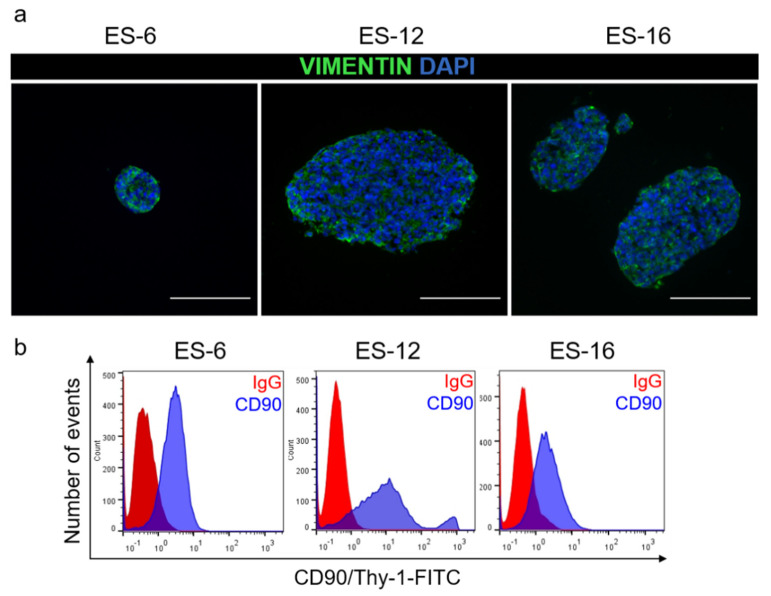
Phenotypic characterization of ES cells after three weeks of encapsulated spheroid culture. (**a**) Immunofluorescence detection of vimentin (green) in encapsulated spheroids generated from three distinct ES-PDX; nuclei were counterstained with DAPI (blue). Scale bar: 100 µm. (**b**) Flow cytometry detection of CD90/Thy-1 (blue) on ES cells retrieved from encapsulated spheroid cultures generated from three distinct ES-PDX. IgG isotype control represented in red.

Having determined that PDX-derived ES cells expanded *in vitro* as encapsulated spheroids retained the main genetic feature of ES cells, we further assessed their phenotype. We analysed the presence of mesenchymal proteins typical of ES cells, such as vimentin and CD90 [37,38,39], as well as ECM components reported to be secreted by ES cells in relevant amounts, such as fibronectin, laminin and collagen [40,41]. After three weeks in culture, vimentin and CD90 were detected by immunofluorescence microscopy and flow cytometry, respectively (Figure 3a,b).

**Figure 4:**
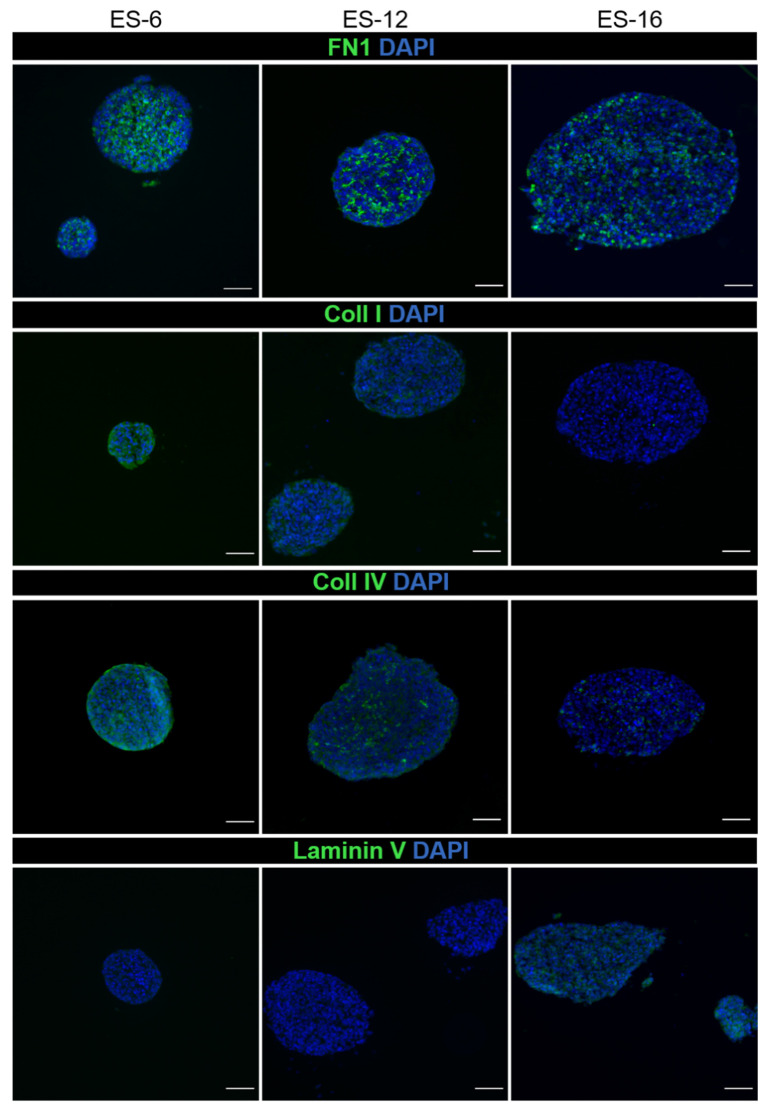
Detection of extracellular matrix proteins after three weeks of encapsulated spheroid culture. Immunofluorescence detection of fibronectin, collagen I, collagen IV and laminin (green) in encapsulated spheroids generated from three distinct ES-PDX; nuclei were counterstained with DAPI (blue). Scale bar: 50 µm.

As for ECM components, fibronectin was detected in the three samples tested, while collagen I and IV and laminin were differentially detected among samples (Figure 4). In ES-12 and ES-16 cells cultured in 2D, fibronectin was also detected (Figure S2).

### 3.4. Proof of Concept of the Applicability of Encapsulated ES Spheroids Cultures in Drug Assays

**Figure 5:**
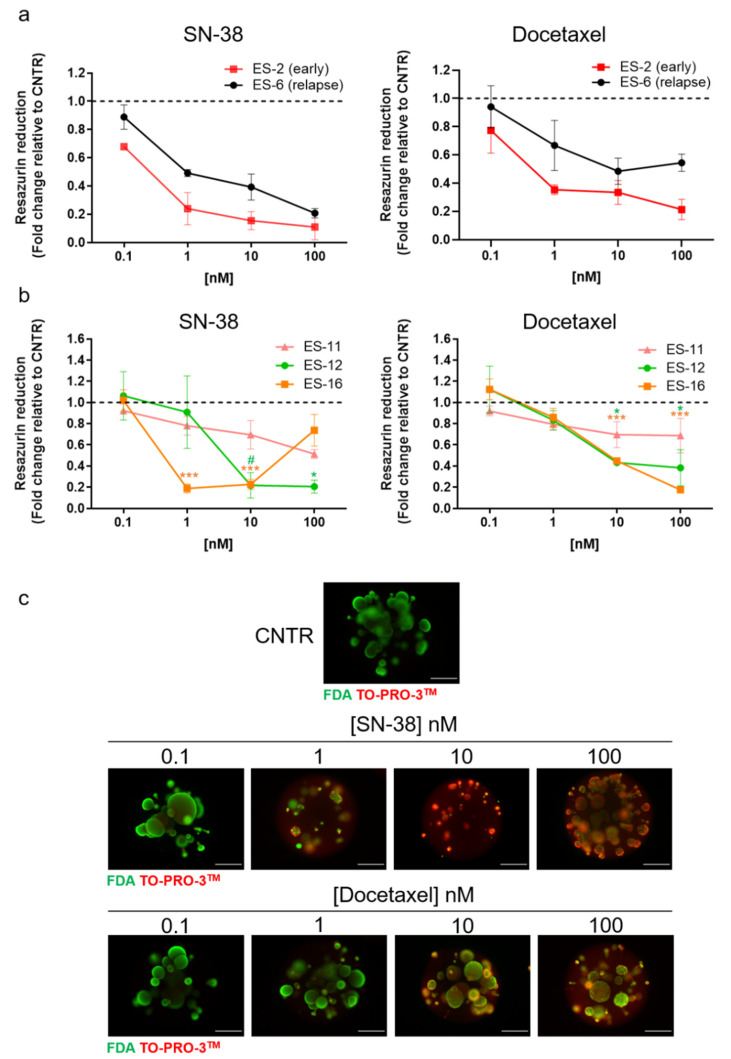
Chemotherapeutic challenge in encapsulated ES spheroid cultures. Resazurin reduction capacity of encapsulated ES spheroid cultures after exposure to SN-38 and docetaxel for seven days: (**a**) ES-2 (*n* = 2) and ES-6 (*n* = 3); (**b**) ES-12 (*n* = 3), ES-16 (*n* = 3) and ES-11 (*n* = 2). Data are represented as the fold change in resazurin reduction of drug-challenged cultures compared to cultures exposed to the drug vehicle control (DMSO; set as 1, dashed line) ± SEM. For ES-12- and ES-16-derived cultures, one-way ANOVA followed by Dunnet’s test were performed, comparing each drug concentration with CNTR (control, vehicle-challenged cultures; # = *p*:0.053, * = *p* < 0.05, *** = *p* ≤ 0.001). (**c**) Representative FDA (live cells, green) and TOPRO-3^TM^ (dead cells, red) staining of encapsulated spheroids cultures derived from ES-6, exposed to increasing concentration of SN-38 and docetaxel. Scale bar: 500 µm.

Having established the culture method and demonstrated that it retains both high cell viability and relevant phenotypical ES markers, we further explored the platform for its applicability in drug assays. As a proof-of-concept, we tested the response of encapsulated ES spheroids to standard-of-care drugs, namely docetaxel [42] and the active form of irinotecan, SN-38 [43,44,45], both used in primary and relapsed ES. After two weeks in culture, we distributed encapsulated and non-encapsulated spheroids into well plates and exposed them to a nanomolar range of concentrations of docetaxel and SN-38, over seven days. Employing cell metabolic status as readout, measured through resazurin reduction activity, we observed that in most of the cases there was a dose response to both drugs in encapsulated ES spheroids (Figure 5a,b). For non-encapsulated spheroids, we observed a reduction in metabolic activity upon SN-38 exposure but not upon docetaxel challenge (Figure S3c–e). In general, responses to both drugs in non-encapsulated ES spheroids were much reduced compared to encapsulated spheroids derived from the same ES-PDX. Cell viability analysis through fluorescence microscopy corroborated the cell metabolism data; we observed an increased detection of TO-PRO-positive cells (dead cells) and a concomitant decrease in detection of FDA-positive cells (live cells) along with the increasing concentration of the drug applied to encapsulated ES spheroid cultures (Figure 5c). For cultures derived from ES-11, -12 and -16, we observed a differential response to docetaxel and SN-38 (Figure 5b), in line with the heterogeneous response to drugs normally observed in patients [45,46,47]. We analysed drug response in cultures derived from the ES-2 and ES-6 PDX, which were established from the same patient at diagnosis and after relapse, respectively. Interestingly, we observed a trend for increased response to both drugs in the early-derived cells versus the relapse-derived cells (Figure 5a), which is in agreement with the in vivo PDX data [48].

To further corroborate the mode of action of SN-38 [49], we analysed *CCND1* and *p21* levels after exposure to the compound. We observed that after SN-38 exposure, *CCND*1 mRNA levels decreased in a time-dependent manner in three out of four samples, while *p21* mRNA level increased in comparison to control in two out of four samples (Figure S3a,b). The ES-11 sample showed no changes in either *p2*1 or *CCND1* mRNA level compared to control upon SN-38 exposure, in line with the general reduced response to SN-38 treatment compared to the other ES samples analysed (Figure 5a,b).

Collectively, these data indicate that the 3D cell culture method proposed here represents a suitable strategy to perform drug assays, enabling repeated cycles of drug challenge with the advantage of reduced contamination with mouse-derived cells compared to regular 2D cell culture.

## 4. Discussion

The limited availability of in vitro models of ES justifies the development of novel methods to study the pathobiology of the disease and to strengthen the drug discovery process, in view of the personalized medicine scenario [50]. ES is a very rare disease and few cell lines are available, even though novel cell lines have been recently established [14]. Besides this, it is well-accepted that 2D in vitro models do not fully recapitulate important aspects of tumourigenesis, such as proliferation rate and response to drugs, including in ES [51]. There is increasing interest in the development of novel 3D in vitro cancer cell models that recapitulate relevant cancer-related biological aspects such as cell-to-cell interaction, TME molecular crosstalk and response to chemotherapy more precisely than 2D cultures [28,29,51,52].

3D cell models can be established through a variety of methods based on static and dynamic techniques [53], as well as by using scaffolds, such as Matrigel, collagen and alginate [26,48]. For instance, the ES cell line TC-71 was grown in 2D as a monolayer, in mice xenografts and in 3D in a porous electrospun poly(ε-coprolactone) scaffold [54]. ES TC-71 cells growing in this scaffold showed IGF-1R/mTOR activation similar to in vivo TC-71 cell line xenografts, which was not observed in cells cultured in 2D. In this report, ES cells cultured in 3D and growing engrafted in mice xenografts showed similar expression of the receptors c-kit and HER2/neu. Finally, cell proliferation inhibition due to doxorubicin in the TC-71 cell line xenografts was reproduced better in the 3D seeded scaffolds than in 2D monolayers. This approach was further refined by coupling flow perfusion to the scaffold in order to modulate IGF-1 secretion by cultured cells as well as to regulate their response to the chemotherapeutic blockage of IGF-1R [55]. Nevertheless, these studies were performed with a single ES cell line and never complemented with primary tumour-derived materials, which, in general, are not easy to obtain due to the rarity of ES cases. PDX models may provide a better source of cells for these studies because initial tumour pieces can be passaged in subsequent mouse generations, enabling long-term in vivo maintenance of the cancer cells transplanted, even in the case of sarcomas [56,57]. As the maintenance of PDX raises ethical and economic concerns, we wanted to determine whether it was possible to maintain ES cells in 3D culture, deriving these cells from ES-PDX explants. In the present report, we illustrated a novel method to maintain ES spheroids derived from established mouse PDX, based on alginate encapsulation. We have previously shown that alginate encapsulation of cancer cell line spheroids co-cultivated with fibroblasts (or both with fibroblasts and blood-derived monocytes) is a powerful and suitable method to mimic cancer-related aspects such as ECM accumulation, endocrine and chemoresistance and macrophage polarization [28,29]. Moreover, high cell viability throughout culture suggested that barium-dependent alginate gelling did not affect ES cell viability, as previously shown also for other cell types [58], indicating that the alginate polymerization methodology is not toxic toward the encapsulated ES cells.

Our results indicate that the 3D conditions applied to ES-PDX-derived cells represent a good method to maintain healthy primary cell cultures for at least one month. For most of the PDX tested, cells remained proliferative during culture time. ES-11-derived spheroids showed reduced cell growth compared with spheroids derived from the remaining ES samples, despite high cell viability, suggesting low cell proliferation levels of ES-11 cells. Interestingly, ES-11 showed a reduced growth in vivo compared to ES-6 [59]. The limit of longevity of these cultures remains to be determined, as repeated drug exposure cycles (even beyond one month) could be required to assess the potential of novel therapeutic modalities. Also, it would be interesting to address the possibility of perpetuating the 3D cultures by passaging the cells from encapsulated spheroids, avoiding the need to restart the 3D-culture setting from PDX-derived cells. This would require optimization of spheroid dissociation conditions.

The confirmation of the expression of *EWSR1-FLI1*, the most relevant protein in the pathogenesis of ES, as well as its target gene *DAX1* [36,60], corroborates the suitability of the culture methods for maintaining the phenotype of the original cancer cells. The maintenance of EWSR1-FLI1 is relevant not only in order to have a tool to study the relevance of the oncogenic contribution of these proteins in ES progression but also as a potential method to assess the potential pharmacological application of EWSR1-FLI1-specific inhibitors [61].

Our observation that the mRNA of the mouse ribosomal protein 36B4 [62] was strikingly reduced in 3D cultures compared to 2D monolayer cultures indicates that the mouse-derived cell contamination is much more amplified in 2D ES cultures compared to 3D ones. This observation can be explained by the ability of fibroblasts to promptly adhere to plastic surfaces [63]. This information suggests that the 3D spheroid cultures are superior to 2D cultures in avoiding mouse-derived cells contamination, which may generate erroneous results (for example, due to increased nucleic acid contamination by mouse DNA/RNA in 2D compared to 3D culture). Furthermore, we observed that 2D cell cultures from these samples were difficult to achieve. In fact, we noticed that single cells plated on regular plastic surfaces required many days before attaching and spreading over the plastic surface (data not shown), as reported also by a pioneer study in the field of ES cell culture [64]. Some PDX-derived cells never attached to the plastic surface and others attached but did not proliferate, raising the possibility that only certain clones of the original plated cell population can be amplified in 2D. We did not passage these cells but used them directly in the assays whenever we could obtain sufficient cell numbers, Nevertheless, we were able to obtain 2D plated cells from four of the ES-PDX (ES-2, -6, -11, -12), which were assessed in parallel with the respective 3D cultures (e.g., to evaluate mouse *36B4* levels). 

An interesting observation was that the ES 3D spheroids did not undergo hypoxia, since we found neither increased mRNA expression of the HIF-1α transcriptional target *CAIX* [35] nor a hypoxic environment detectable by a specific hypoxia fluorescent dye. Hypoxic cores in cancer cell spheroids have been observed only when the spheroid reached a diameter of more than 400 μm [65], whereas the mean Feret’s diameter of encapsulated spheroids was much lower, between 127.43 μm (ES-12) and 156.6 μm (ES-16). Moreover, oxygen can diffuse freely within alginate capsules [66]. These observations suggest that in order to use this 3D model to study ES cell behaviour/cell response under hypoxia, exposure to a hypoxic environment is required [67,68]. The methodology proposed here, applying chemically or environmentally hypoxic conditions, can then be employed to investigate the role of a hypoxia microenvironment in ES, since it has been shown that hypoxic conditions induce EWSR1-FLI1 expression in ES cells [67].

ES, as well as other sarcomas, are normally not infiltrated by fibroblasts [69]. These cells are typically involved in the production of ECM components within TME, and it is known that ES cells may themselves produce ECM proteins [41]. For instance, fibronectin is secreted by many ES cell lines such as A4537, 6647, TC106 and 5838 [41] and we detected it also in 2D cultures, indicating that our cultures shared this characteristic with established ES cell lines. Fibronectin detection in ES was also confirmed by a proteomic study of the secretome of ES cells TC-32 and CHLA10, which revealed that fibronectin was one of the most abundantly secreted proteins [40]. Fibronectin was also clearly detected in osteosarcoma cells cultured in 3D [70]. Thus, 3D models could be useful to understand the role of fibronectin in ES, which remains unknown.

Finally, our assay with cytotoxic drugs demonstrated that this 3D platform is useful to test the effect of novel drugs against ES. We observed a milder response to SN-38 in encapsulated spheroid cultures derived from a PDX originating in a relapse (ES-6) compared to those derived from a PDX of the same patient at diagnosis (ES-2), recapitulating the drug sensitivity data obtained in PDX [48]. Strikingly, tumour relapse was much more enhanced in irinotecan-treated ES-2 mice (derived at diagnosis) than in ES-6 PDX (derived from late-relapse sample), in line with the trend we observed in encapsulated ES spheroids. In non-encapsulated spheroids, the responses were generically milder, which can be potentially explained by reduced drug penetration in larger cell clusters formed by spheroids.

The superiority of 3D platforms over 2D cell cultures has also been suggested by previous studies. A previously developed ES cell line-based 3D cell model showed similar constitutive activation of signalling pathways as IGF-1R and mTOR in a 3D scaffold as observed in vivo, as well as higher chemotherapeutic resistance when compared to 2D monolayers, mimicking human ES response in vivo [54]. Another 3D cell model (based on 3D porous matrices composed by collagen I and hyaluronan, coupled to mechanical stimulation) was proposed to study the biomechanical regulation of drug sensitivity in ES cell lines and PDX-derived cells [39]. In this report, the authors showed the biomechanical reactivation in 3D of signalling pathways know to be active in vivo, such as ERK1/2, but inactive in 2D cultures. The platform we developed here, based on PDX-derived cells, is able to maintain high cell viability and intrinsic ES characteristics, such as the EWSR1-FLI1 translocation and ECM secretion, as well as providing a tool to analyse the chemotherapeutic response to proof-of-concept drugs. The presence of ECM makes the model even more interesting for its relevance in term of drug response, as it has been shown that ECM secretion and, more generally, the TME, may alter the response to chemotherapeutic drugs [71,72]. Altogether, our findings, along with previous observations, endorse a more extensive use of 3D cell models in ES biomedical research to better recapitulate TME aspects in vitro.

## 5. Conclusions

In conclusion, we showed that it is possible to culture ES-PDX derived cells as spheroids and that, employing alginate microencapsulation and agitation-based culture, spheroids can be maintained in culture for at least one month, with high cell viability, while preserving their mesenchymal nature and ECM expression. Importantly, the culture method presented here has the advantage of maintaining *EWSR1-FLI1* translocation expression and reducing the amount of mouse contaminating cells compared to 2D cultures, making it an optimal methodology to study ES pathobiology itself and in relation to EWSR1-FLI1. This methodology may serve also as a new tool in the drug discovery process. Finally, taking into account that we have previously shown that it is possible to co-culture different cell types within alginate capsules [29], we envisage that it will be possible to expand the application of this culture methodology to more complex cellular systems. For example, we believe that ES cells co-cultured with other cell types, such as leukocytes, will help further understand the ES immune environment. In fact, it has been shown that ES can be infiltrated by T-lymphocytes [73] and macrophages [74], which can affect patient outcomes, thus highlighting the need for the study of ES cells with other relevant tumour microenvironment cell types.

## Data Availability

The data presented in this study are available in the article or supplementary files.

## Supplementary Materials

The following are available online at https://www.mdpi.com/2072-6694/13/4/879/s1, Figure S1: ES spheroid cell viability and proliferation in culture, Figure S2: Fibronectin (FN1) IF staining in ES-12 and ES-16 cultured in 2D. Figure S3: *CCND1* and *p21* expression in encapsulated ES spheroids and drug response in non-encapsulated spheroids, Figure S4: Uncropped and unadjusted agarose gel image relative to Figure 2A.
