# Supplementary material for: PDX-Derived Ewing’s Sarcoma Cells Retain High Viability and Disease Phenotype in Alginate Encapsulated Spheroid Cultures
Source: Cancers (Basel). 2021 Feb 19;13(4):879. doi: 10.3390/cancers13040879 (PMC7922076; doi:10.3390/cancers13040879)
Supplement: Supplementary file 1 [file cancers-13-00879-s001.pdf]

cancers

MDPI

Supplementary Materials: "PDX-Derived Ewing's Sarcoma Cells Retain High Viability and Disease Phenotype in Alginate-Encapsulated Spheroid Cultures"

Giacomo Domenici, Rodrigo Eduardo, Helena Castillo-Ecija, Gorka Orive, Ángel Montero Carcaboso and Catarina Brito

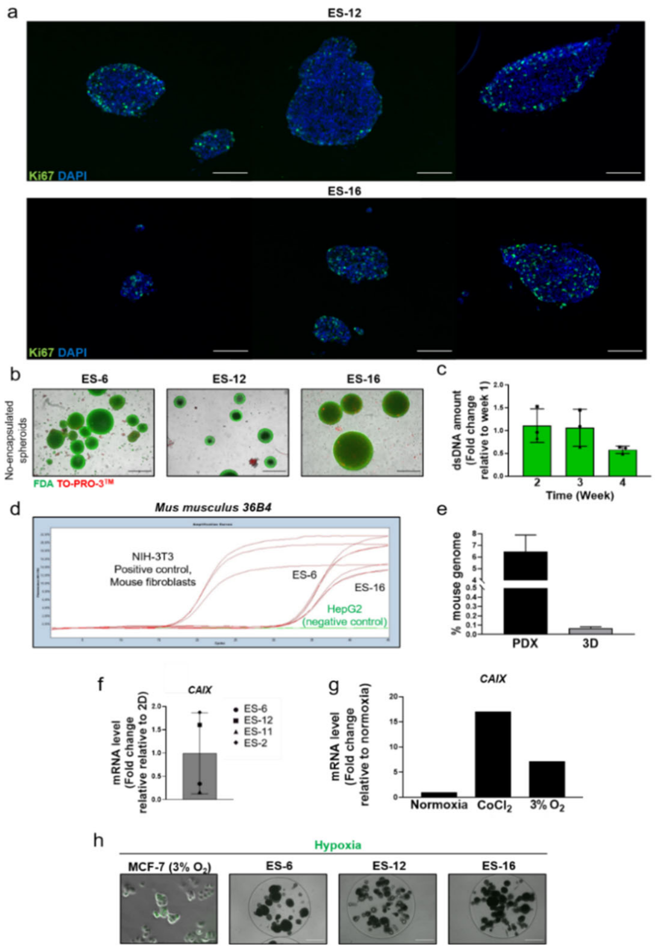

Figure S1. Cell viability and proliferation in ES spheroid cultures (**a**) Immunofluorescence detection of Ki67 (green) in encapsulated ES spheroids; nuclei were counterstained with DAPI (blue). Scale bar: 100 µm. (**b**) Representative images of non-encapsulated spheroid cultures derived from ES-6, ES-12 and ES-16. Live cells (FDA, green) and dead cells (TO-PRO-3™). Scale bar: 500 µm. (**c**) dsDNA amount along non-encapsulated spheroid cultures derived from 3 distinct ES-PDX, shown as fold change at each culture time relative to week 1 (set as 1, dashed line). Statistical analysis was performed by the non-parametric Kruskal Wallis test. (**d**) Representative RT-qPCR amplification curves of 36B4 mRNA in mouse NIH-3T3 cells, encapsulated spheroid cultures (derived from ES-6 and ES-16) and human hepatocarcinoma cell line HepG2. (**e**) Percentage of mouse genome in encapsulated spheroid cultures derived from ES6 and ES12 after one-month culture relative to the original PDX cells ($n = 2$)**.** (**f**) Fold change in mRNA expression levels of CAIX in in 2D monolayer and encapsulated spheroid cultures derived from 4 different ES-PDX ($n = 4$). (**g**) MCF-7 cells were exposed to 800 µm $CoCl_2$ (in normoxia) or to 3% O_2, for 24 h. Data are presented as fold change in mRNA level in each condition relative to normoxia control condition. (**h**) Environmental hypoxia analysis by Image-iT™ Green Hypoxia Reagent in encapsulated ES spheroid cultures and control MCF-7 2D monolayers exposed to 3% O_2. MCF-7 cells: scale bar: 50 µm; encapsulated ES spheroids: scale bar: 500 µm.

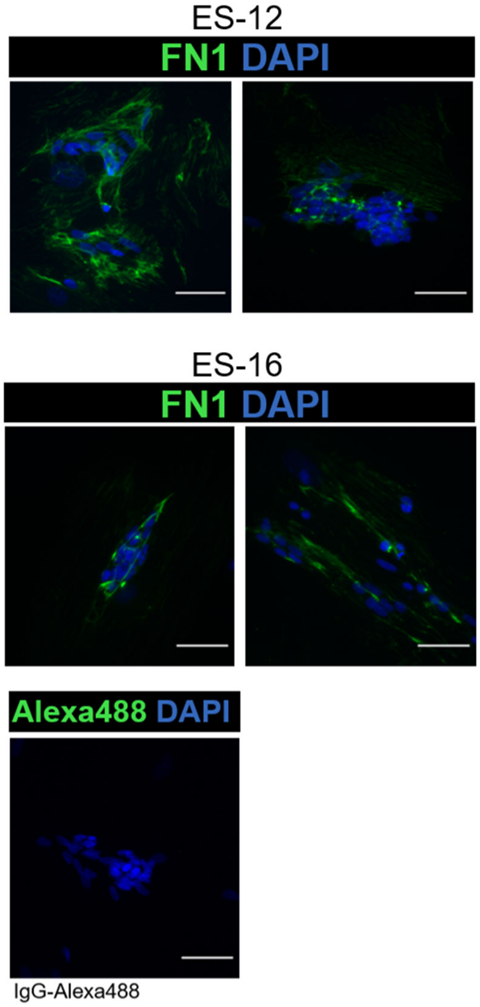

Figure S2. Detection of fibronectin (FN1) in ES12 and ES16-derived 2D cultures. Nuclei were counterstained with DAPI. Secondary antibody control (IgG-Alexa488) is shown as well. Scale bar: 50 μm.

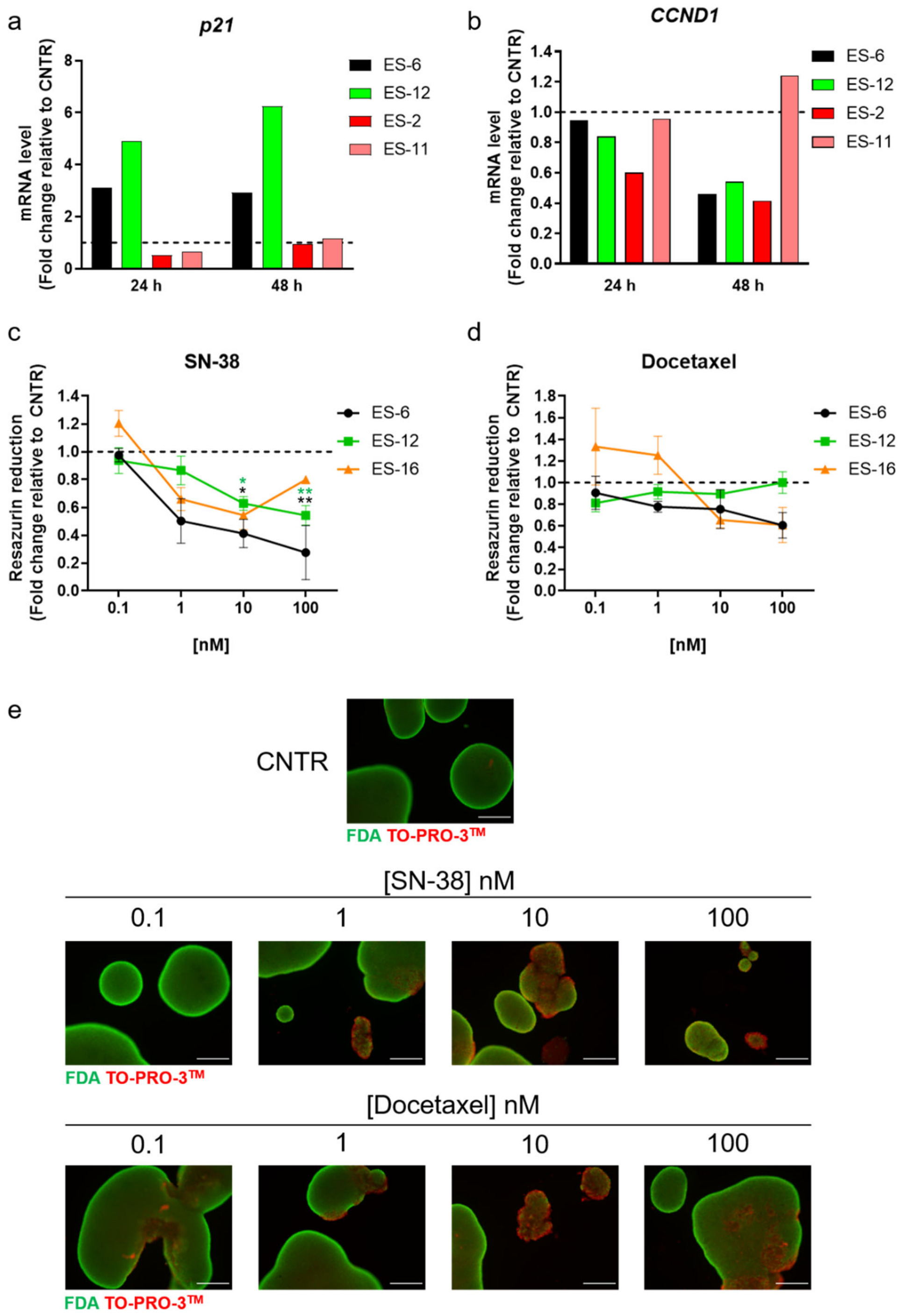

Figure S3. *CCND1* and *p21* expression in encapsulated ES spheroids and drug response in non- encapsulated spheroids (**a**) *CCND1* mRNA expression in encapsulated ES spheroid cultures exposed to 10 nM SN-38, for 24 or 48 h. Data are shown as fold change in *CCND1* mRNA level relative to vehicle control (DMSO; set as 1, dashed line). (**b**) *p21* mRNA expression in encapsulated ES spheroid cultures exposed to 10 nM SN- 38, for 24 or 48 h. Data are shown as fold change in *p21* mRNA level relative to vehicle control (DMSO; set as 1, dashed line). (**c**) Resazurin reduction in non-encapsulated ES spheroid cultures, exposed to SN-38 for 7 days (ES-6, -12: *n* = 3; ES-16: *n* = 2). (**d**) Resazurin reduction in non-encapsulated ES spheroid cultures exposed to Docetaxel (ES-6, -12: *n* = 3; ES-16: *n* = 2). Data are shown as fold change in resazurin reduction relative to vehicle control (DMSO; set as 1, dashed line) ± SEM. For ES-12 and ES-16 derived cultures, one-way ANOVA followed by Dunnet's test was performed, comparing each drug concentration with CNTR,(control, vehicle-challenged

cultures * = p <0.05, ** = $p \leq 0.01$). (**e**) Representative FDA (live cells, green) and TOPRO-3™ (dead cells, red) staining of encapsulated spheroid cultures derived from ES-6, exposed to increasing concentration of SN-38 and Docetaxel. Scale bar: 500 µm.

Figure 2A Gel

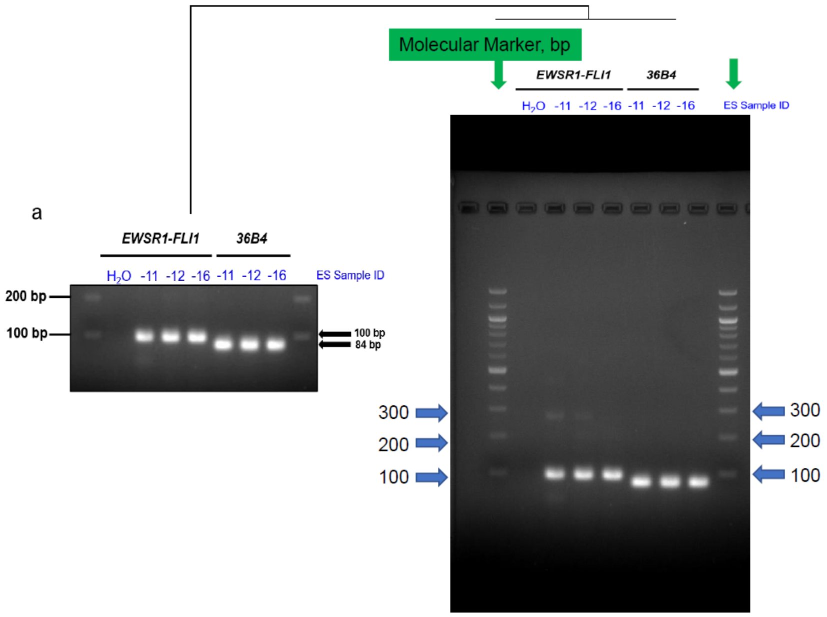

Figure S4. Uncropped and unadjusted agarose gel image relative to Figure 2A. bp: base pair.

Table S1. Characterization of PDX models.

Pdx Model	Patient	Disease Stage	Translocation	Time to Endpoint [b] (day)	Pdx Passage in Mice
HSJD-ES-002	1	Diagnosis [a]	*EWSR1-FLI1*, ex10-ex5	52	6
HSJD-ES-006	1	Relapse	*EWSR1-FLI1*, ex10-ex5	46	4
HSJD-ES-011	2	Relapse	*EWSR1-FLI1*, ex7-ex6	62	3
HSJD-ES-012	3	Relapse	*EWSR1-FLI1*, ex7-ex6	94	5
HSJD-ES-016	4	Relapse	*EWSR1-FLI1*, ex7-ex6	29	9

[a] No previous treatment at the time of the biopsy, [b] Defined as the time to achieve 1500 mm^3.

Table S2. Primer List.

Gene	Forward 5′-3′	Reverse 5′-3′
EWSR1	CCAAGTCAATATAGCCAACAG	-
FLI-1	-	GGCCAGAATTCATGTTATTGC
36B4	GTGTTCGACAATGGCAGCAT	GACACCCTCCAGGAAGCGA
RPL22	CACGAAGGAGGAGTGACTGG	TGTGGCACACCACTGACATT
Mouse *36B4*	TGCTCGACATCACAGAGCAG	ACGCGCTTGTACCCATTGAT
CCND1	AGACCTTCGTTGCCCTCTGT	TGTGAGGCGGTAGTAGGACA
p21	AGTCAGTTCCTTGTGGAGCC	GCATGGGTTCTGACGGACAT

CAIX	TTTGCCAGAGTTGACGAGGC	TCTTCCAAGCGAGACAGCAAC
DAX1	GGGGACCGTGCTCTTTAACC	GCGTCATCCTGGTGTGTTCA
Mouse *PTGER2*	CCTGCTGCTTATCGTGGCTG	GCCAGGAGAATGAGGTGGTC
Human *PTGER2*	GCTGCTTCTCATTGTCTCGG	GCCAGGAGAATGAGGTGGTC
